# How does chemotherapy treatment damage the prepubertal testis?

**DOI:** 10.1530/REP-18-0221

**Published:** 2018-10-12

**Authors:** Caroline M Allen, Federica Lopes, Rod T Mitchell, Norah Spears

**Affiliations:** 1 Biomedical Sciences, University of Edinburgh, Edinburgh, UK; 2 MRC Centre for Reproductive Health, University of Edinburgh, Edinburgh, UK

## Abstract

Chemotherapy treatment is a mainstay of anticancer regimens, significantly contributing to the recent increase in childhood cancer survival rates. Conventional cancer therapy targets not only malignant but also healthy cells resulting in side effects including infertility. For prepubertal boys, there are currently no fertility preservation strategies in use, although several potential methods are under investigation. Most of the current knowledge in relation to prepubertal gonadotoxicity has been deduced from adult studies; however, the prepubertal testis is relatively quiescent in comparison to the adult. This review provides an overview of research to date in humans and animals describing chemotherapy-induced prepubertal gonadotoxicity, focusing on direct gonadal damage. Testicular damage is dependent upon the agent, dosage, administration schedule and age/pubertal status at time of treatment. The chemotherapy agents investigated so far target the germ cell population activating apoptotic pathways and may also impair Sertoli cell function. Due to use of combined chemotherapy agents for patients, the impact of individual drugs is hard to define, however, use of* in vivo* and *in vitro* animal models can overcome this problem. Furthering our understanding of how chemotherapy agents target the prepubertal testis will provide clarity to patients on the gonadotoxicity of different drugs and aid in the development of cytoprotective agents.

## Introduction

The overall childhood cancer survival rate has increased substantially in recent decades, with the current 5-year survival rate at around 80%, compared to about 58% in the late 1970s (Miller *et al*. 2016). This marked advance, due in large part to improved chemotherapy treatments, has led to a growing population of long-term childhood cancer survivors. However, chemotherapy drugs do not exclusively target malignant cells, also eliciting side effects due to off-target damage to healthy tissues. Given this situation, research is increasingly focusing on preventing damage to healthy organs, to improve the quality of life for childhood cancer survivors. For younger patients, detrimental effects of treatment on fertility can be a major concern (Zebrack *et al*. 2004). This is particularly problematic for male survivors of childhood cancer, given the current lack of available fertility preservation treatments, since, unlike adult patients, these prepubertal patients do not yet produce mature spermatozoa that can be used for routine sperm cryopreservation (Wyns *et al*. 2010, Wallace 2011, Mitchell *et al*. 2017). Recently, centres have started to cryopreserve immature testicular tissue from prepubertal boys before the commencement of chemotherapy treatment. In 2015, there were seven centres in Europe collecting such tissues with more than 260 prepubertal samples stored, with biopsies undertaken only when treatment is deemed at high risk for later fertility complications (Picton *et al*. 2015, Mitchell *et al*. 2017). At this point, however, it is not yet certain if such cryopreserved tissue can be successfully used later to restore fertility in humans, as production of viable sperm from such tissue is yet to be shown. A recent report has described the generation of sperm-like cells after 3D culture of isolated spermatogonial cells obtained from testis biopsies taken from prepubertal boys undergoing chemotherapy treatment (Abofoul-Azab *et al*. 2018). This is encouraging for the future, but in this preliminary study, the technique was successful in only one out of six patient biopsies, and the functionality of the sperm-like cells remains to be established. Research in animal models has been more successful in showing the potential of fertility preservation techniques, which could be developed for clinical use (reviewed in Picton *et al*. 2015, Giudice *et al*. 2017). Transplantation of spermatogonial stem cells (SSCs) or frozen-thawed immature testicular tissue grafted back into the adult has been successful in producing functional gametes in animal models including the non-human primate and murine (Brinster & Zimmermann 1994, Mitchell *et al*. 2010, Hermann *et al*. 2012, Jahnukainen *et al*. 2012). Healthy offspring have been produced through IVF/ICSI using sperm derived from xenografted non-human primate immature testicular tissue demonstrating the potential of this technique for clinical application (Liu *et al*. 2016). There are concerns, however, for non-solid tumours that malignant cells could be reintroduced back into the patient, particularly with tissue transplantation as shown in the Hou *et al*. (2007) study where leukaemic infiltration was noted within the xenografted testicular tissue. In addition to *in vivo* techniques, sperm has been grown in culture from immature testis through *in vitro* spermatogenesis, and these sperm have been used for IVF/ICSI to produce viable embryos in a mouse model system (Sato *et al*. 2011). There have also been recent reports of *in vitro* culture of human prepubertal testicular tissue, although without completion of spermatogenesis (de Michele *et al*. 2017, 2018). For all such potential fertility preservation techniques, further research is needed to allow the methods to be successfully transformed for use with human tissue, after which further validation will be required to ensure that such methodologies are efficient and safe for clinical use. An alternative strategy, rather than the subsequent restoration of fertility after chemotherapy-induced infertility, would be the development of interventions administered before and/or during chemotherapy treatment to prevent the damage from occurring in the first instance, thus protecting the fertility potential of the patient. Cytoprotective agents that specifically protect the prepubertal testis against chemotherapy-induced damage, without interfering with the toxicity to cancer cells, could potentially be employed as part of the chemotherapy treatment regimen. However, further research is required to fully understand how chemotherapy drugs target the prepubertal testis and which compounds could potentially prevent such damage.

This review provides an overview of research to date that has focused upon the direct, chemotherapy drug-induced damage to the prepubertal testis: such studies often rely on histological analysis. Damage can also be conferred from studies examining hormonal changes after cancer treatment. For example, AMH and inhibin B can be used as markers of Sertoli cell function, although the use of such markers during prepuberty is yet to be investigated (Dere *et al*. 2013, Stukenborg *et al*. 2018*a*). The use of hormonal indicators to determine chemotherapy-induced damage is not discussed further in this review. The review discusses papers only where they investigate effects on the prepubertal testicular tissue/cells, either from human or animal models. Determining which cell types within the prepubertal testis are directly targeted by chemotherapy drugs, along with the potential mechanism of action of the different drugs, will provide vital information enhancing our knowledge on the gonadotoxicity of chemotherapy agents and the development of cytoprotective agents against chemotherapy-induced damage.

## Methodology

**Figure 1:**
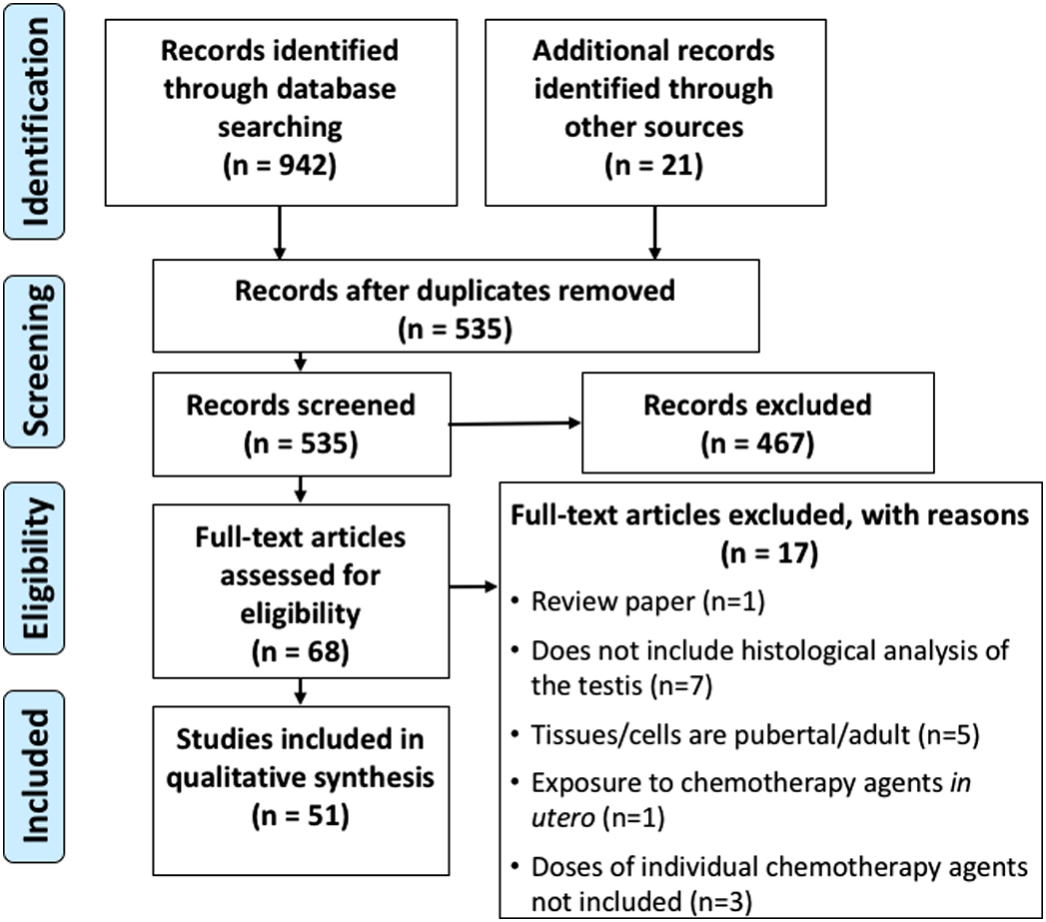
PRISMA flow diagram of literature search. PRISMA flow diagram of search results, study screening, and study inclusion, following a review of the literature carried out using PRISMA guidelines (Moher *et al*. 2009).

A literature search was conducted to identify relevant papers to query our research question; how does chemotherapy treatment damage the prepubertal testis? A review of the literature was performed using PRISMA guidelines (Moher *et al*. 2009). Relevant papers were identified using PubMed to search for appropriate references, searching key words including prepubertal/immature, testes, chemotherapy (including classes of drugs) and fertility preservation. Additional references were found by searching reference lists of such papers. The abstracts of identified papers were screened for relevance in relation to chemotherapy treatment during prepuberty and impact on the testis. The eligibility of relevant studies was assessed by reading the screened papers in full to ensure that all those included in this review are all concerning prepubertal tissues, whether from human patients or animal models. Clinical studies are included where the histology of the testis of human patients following prepubertal cancer treatment is examined during or at the end of the treatment period or during adulthood. *In vivo* studies in animal models where drug exposure occurred prior to the onset of puberty as well as *in vitro* studies of cultured cells and tissues obtained from prepubertal animals were also analysed. Potential fertility cytoprotectants were included where research was performed on prepubertal/immature subjects. Of the papers that were excluded, the majority were due to chemotherapy treatment taking place during/after puberty, analysis of chemotherapy-induced damage through hormonal changes or failure to report the dose of the chemotherapy agents. Overview of our research strategy is shown in Fig. 1.

## Childhood cancer and treatment

### Incidence of childhood cancer

Each year around 1800 children are diagnosed with neoplastic disease, accounting for 1% of new cancer diagnoses in the United Kingdom (Wallace 2011, Cancer Research UK 2015). Cancer is the second most common cause of death in children. The nature of cancers that develop at this young age differs from those of adult malignant tumours. In infants, tumours of embryonal origin such as neuroblastomas are the most frequent cause, whereas in older children, leukaemia (particularly acute lymphoblastic leukaemia) as well as central nervous system tumours and lymphomas (non-Hodgkins and Hodgkins) are more often diagnosed. There is also a sex difference in incidence, with boys 1.2 times more likely to be diagnosed with cancer, particularly lymphomas and central nervous system tumours (Kaatsch 2010, Kelly 2017).

### Chemotherapy treatment

Chemotherapy and radiotherapy are a mainstay of treatment for childhood malignancies. For chemotherapy drugs, the refinement and use of combined drug treatments have contributed greatly to the dramatic increase in childhood cancer survival rates over recent decades (Anderson *et al*. 2015, Miller *et al*. 2016). Multiple different drugs have been established in treatment regimens; these kill cancer cells through various mechanisms, often targeting proliferating cells (Table 1) (Malhotra & Perry 2003, Lind 2011). The gonadotoxic impact and relative risk of later infertility of individual chemotherapy agents included in treatment regimens against childhood malignancies can at present only be estimated, since the risk is dependent on multiple factors including dosage, treatment length, age at time of treatment and the sensitivity of individual patients to chemotherapy treatment. The current classification of chemotherapy drugs and the dosages considered to result in infertility are outlined in Table 1; however, these classifications/dosages are debated. Given that clinicians use this knowledge to determine whether or not to offer cryopreservation of testicular tissue before commencement of treatment, further elucidation on relative risk/gonadotoxicity is urgently required. At present, alkylating and alkylating-like agents such as cyclophosphamide and cisplatin are considered to be highly gonadotoxic with these agents intercalating into DNA, disrupting basic cellular processes. These agents are commonly included in treatment regimens to treat a wide range of childhood malignancies and are known to result in subsequent infertility in adulthood (Chow *et al*. 2016). Agents from alternative drug classes which have different mechanisms of action are considered low or moderate risk of later infertility and include; antimetabolites such as cytarabine, vinca alkaloids e.g. vincristine and topoisomerase inhibitors including etoposide. There are also some chemotherapy agents, including taxanes, where infertility risk/gonadotoxicity is at present unknown. Further details are included in Table 1.

**Table 1:** Overview of commonly used chemotherapeutic agents in childhood cancer treatment regimens.

Chemotherapy drug class	Childhood cancer usage	Mechanism of action	Cell cycle phase	Compounds	Current prediction of infertility risk*
Alkylating and alkylating-like agents	Bone cancerCNS tumoursHodgkins lymphomaKidney cancerLeukaemiaNeuroblastomaNon-Hodgkins lymphomaSoft tissue sarcoma	Alkyl groups intercalate into nucleic acids and proteins. Intercalate into DNA by binding to the guanine or cytosine bases, resulting in crosslinks which disrupt DNA replication/transcription	Non-specific	CarboplatinChlorambucilCisplatinCyclophosphamide Ifosfamide MechlorethamineMelphalanOxaliplatinProcarbazine	ModerateHigh (>1.4 g/m^2^)High (>0.6 g/m^2^)High (>7.5 g/m^2^)High (>60 g/m^2^)HighHigh (0.14 g/m^2^)ModerateHigh (>4 g/m^2^)
Anthracyclines	Bone cancer Hodgkins lymphomaKidney cancerLeukaemiaNeuroblastomaNon-Hodgkins lymphomaSoft tissue sarcoma	Target topoisomerase II, intercalate into DNA and produce free radicals	M-phase	DaunorubicinDoxorubicin	UnknownModerate
Antimetabolites	Bone cancerLeukaemiaNon-Hodgkins lymphoma	Disrupt DNA/RNA synthesis	S-phase	CytarabineFluorouracilMercaptopurineMethotrexateThioguanine	ModerateUnknownLowLowUnknown
Non-Anthracycline Antibiotics	Bone cancerHodgkins lymphomaSoft tissue sarcoma	Multiple mechanisms of action including DNA intercalation, disruption of DNA synthesis and DNA fragmentation	Non-specific	BleomycinDactinomycinMitomycin	LowLowUnknown
Taxanes	Ewing sarcoma	Inhibit disassembly of microtubules	G_2_/M-interphase	DocetaxelPaclitaxel	UnknownUnknown
Topoisomerase inhibitors	Bone cancer LeukaemiaSoft tissue sarcoma	Target either topoisomerase I or II to disrupt DNA replication	M or S-phase	EtoposideIrinotecanTeniposideTopotecan	LowUnknownUnknownUnknown
Vinca alkaloids	Bone cancerCNS tumourHodgkins lymphomaKidney cancerLeukaemiaNeuroblastomaNon-Hodgkins lymphomaSoft tissue sarcoma	Inhibit assembly of microtubules	M-phase	VinblastineVincristine	LowLow

*Currently considered risk of infertility adapted from Wyns *et al.* (2010).

For the vast majority of paediatric cancers, combined chemotherapy with multiple agents is required to effectively treat the disease, with commonly used combinations including MOPP (nitrogen mustard, vincristine, procarbazine and prednisolone) or ABVD (doxorubicin, bleomycin, vinblastine and dacarbazine) for treatment of Hodgkins lymphoma and CHOP (cyclophosphamide, doxorubicin, vincristine and prednisolone) for non-Hodgkins lymphoma (Corrie 2011). The administration of several agents in a treatment regimen could potentially result in additive or even multiplicative side effects on healthy tissues.

## Testis development

**Figure 2:**
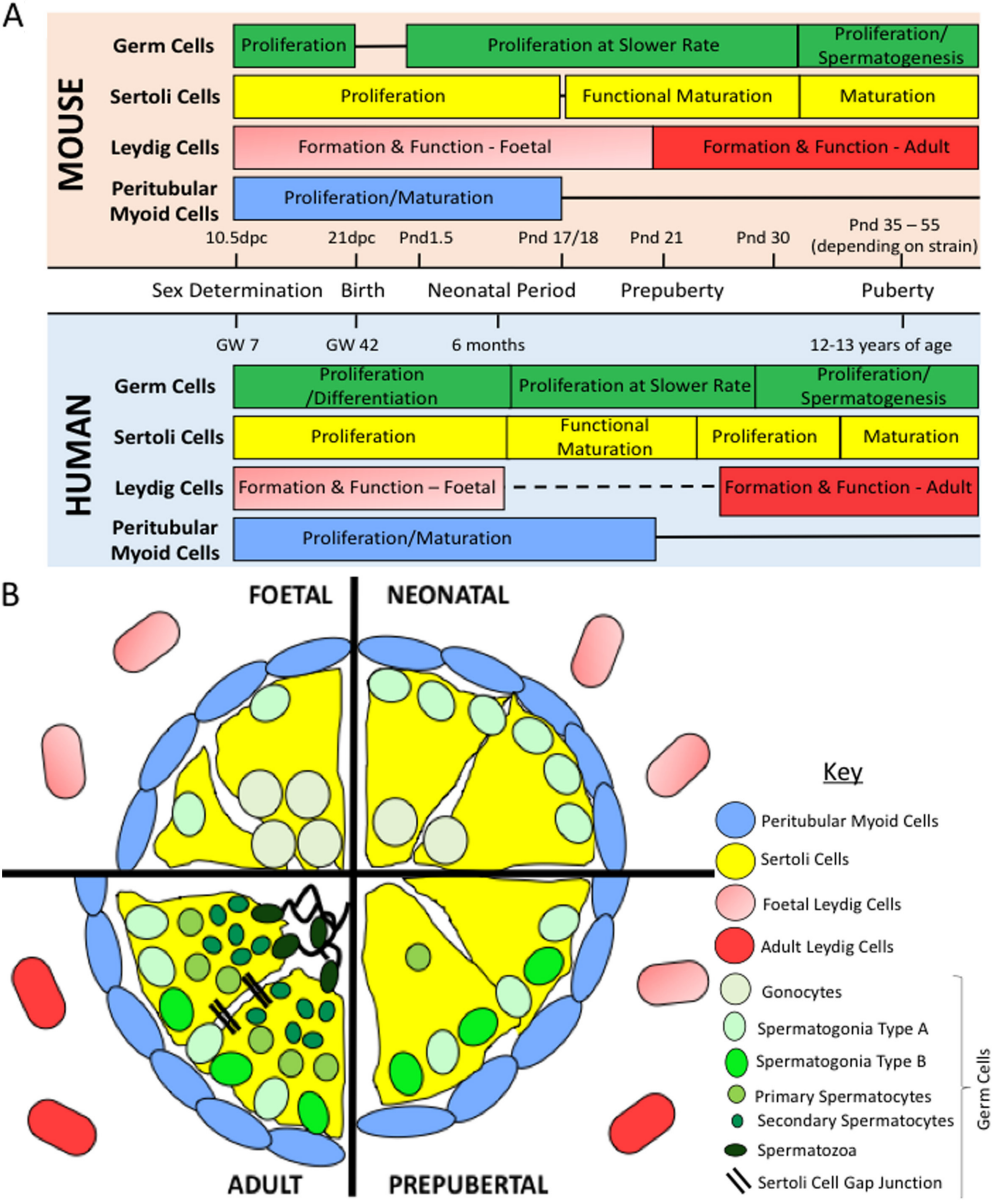
Comparison of testicular development in humans and rodents. (A) Relative timeframe of important developmental processes taking place between foetal development and puberty in humans (Chemes 2001) and the mouse model (Vergouwen *et al.* 1993). Solid line indicates no activity of the cells at the relevant time points and dashed line represents the unknown nature of Leydig cell development during this timeframe. (B) Comparison of the histology of the testis throughout development in the human, from foetal development through to the adult testis. dpc, days post coitum; GW, gestational week; pnd, postnatal day.

The testis is responsible for producing mature spermatozoa along with the main male reproductive hormone, testosterone. During prepuberty, the testis was originally thought to be relatively inactive based on studies demonstrating few morphological changes and a lack of hormone production during this period (Rey 1999). However, further detailed analysis has shown that the prepubertal testis undergoes important developmental processes, which are required for normal adult functioning (Fig. 2A). This section will outline what is currently known regarding testis development, focusing primarily upon human development. Many studies, however, have relied upon animal models to observe prepubertal changes due to the challenges of studying the human testis; information about non-human species will be specified where relevant.

### Foetal life

The testis forms during early foetal life from an undifferentiated bipotential gonad. The primordial germ cells, originally located outside the embryo within the yolk sac, migrate and populate the gonadal ridge (Stukenborg *et al*. 2014). Under the action of the *SRY* (sex-determining region Y) gene, which drives production of the SOX9 (SRY-box 9) protein, Sertoli cells differentiate from precursors cells within the gonadal ridge and engulf the primordial germ cells which are now classified as gonocytes. This configuration results in the formation of seminiferous cords, which predominantly contain Sertoli cells with centrally located gonocytes. The Sertoli cells are key drivers in the differentiation and function of other cellular components of the testis including the Leydig cells and peritubular myoid cells (reviewed in Svingen & Koopman 2013) (Fig. 2B). The hypothalamic–pituitary–gonadal axis (HPG) is active during foetal life, producing the gonadotrophins follicle-stimulating hormone (FSH) and luteinizing hormone, which are essential for the formation, maturation and function of key somatic cells including the Sertoli and Leydig cells (Stukenborg *et al*. 2018*a*).

During foetal development, the gonocytes actively proliferate into early neonatal life, and differentiation of the gonocytes into pre-spermatogonia occurs asynchronously during foetal and early postnatal life, with the gonocytes having greater proliferative activity than the pre-spermatogonia. In the rodent model, however, the proliferation of the gonocytes ceases during late foetal life and resumes during the early postnatal period (Ferrara *et al*. 2006, Mitchell *et al*. 2008, Wu *et al*. 2009). Sertoli cells actively proliferate during foetal development under the control of multiple hormones, particularly FSH. This period of proliferation is very important in determining adult numbers of Sertoli cells (Sharpe *et al*. 2003). Peritubular myoid cells develop from interstitial cells, when multiple layers of these cells along with extracellular matrix proteins surround and form the wall of the seminiferous cords. These cells contribute to the basement membrane providing structural support during testis development and will function in the adult to aid movement of mature sperm towards the lumen (Mayerhofer 2013, Svingen & Koopman 2013). The endocrine somatic Leydig cells are situated within the interstitial compartment between seminiferous cords and are composed of different cell populations throughout an individual’s lifespan. The foetal Leydig cells (FLCs) produce testosterone throughout foetal life, which is essential for masculinization of the male reproductive system. These cells ultimately regress after birth and are replaced by adult Leydig cells (ALCs) during puberty (Habert *et al*. 2001, Chen *et al*. 2009, Svechnikov *et al*. 2010), however, the presence and/or function of the FLCs have recently been shown to be important in the formation and function of the ALCs in a rodent model (Su *et al*. 2018). Whether FLCs and ALCs are distinct populations of cells or share a common stem/progenitor cell lineage is at present unknown; however, stem ALCs have been observed in the human foetal testis (Kilcoyne *et al*. 2014, Teerds & Huhtaniemi 2015).

### Neonatal life

During the first 3 months of neonatal life, the reproductive system undergoes a short period of activity known as ‘mini-puberty’, where the HPG axis is active (Kuiri-Hänninen *et al*. 2014). The origin of the Leydig cells that produce testosterone during ‘mini-puberty’ is unknown, possibly forming from precursor cells or from the FLCs (Svechnikov *et al*. 2010). The function of ‘mini-puberty’ is yet to be clarified, but is thought to be linked to later reproductive function in the adult (Copeland & Chernausek 2016). This short neonatal activity has also been observed in the primate animal model during neonatal development but does not appear to occur in rodents, where infancy overlaps with early stages of pubertal development (Chemes 2001, Kelnar 2002).

The volume of the testis increases six-fold during neonatal development due to proliferation of Sertoli cells stimulated by FSH secretion; 93–95% of the seminiferous cord mass is attributed to the Sertoli cells at this stage (Müller & Skakkebaek 1983, Rey *et al*. 1993; reviewed in Petersen & Söder 2006). This increase in cell number results in an overall increase in seminiferous cord length but not diameter, as each Sertoli cell maintains contact with the basement membrane, the cells spreading lengthwise (Chemes 2001). Therefore, in a cross-sectional area of the seminiferous cord, it may appear as though the cell density remains stable or even decreases during this period of development, which can be misleading. The resulting number of Sertoli cells will ultimately determine sperm production in the adult, with each Sertoli cell able to support a species-specific number of germ cells (Sharpe 2001).

During the foetal and early postnatal period of development, centrally located gonocytes within the seminiferous cords differentiate and lose their pluripotency. At around 2–3 months of age, the differentiating cells migrate towards the basement membrane, developing into the SSCs (Culty 2009). The SSCs are now located within the stem cell niche in the basal compartment of the seminiferous cords, outside of the blood–testis barrier (BTB), which will later form during puberty to protect post-meiotic germ cells (Stanton 2016, Li *et al*. 2017). The maintenance of the SSC niche is dependent on factors produced by somatic cells (Stukenborg *et al*. 2018*a*). In rodents, there is a relatively rapid and synchronous differentiation of gonocytes to pre-spermatogonia and migration to the basal membrane occurring by postnatal day (pnd) six (Mitchell *et al*. 2008, Wu *et al*. 2009).

### Prepuberty

Prepuberty lasts for around 11 years until the HPG axis is once again reactivated and the child enters puberty. During prepuberty, perceived reduced cellular activity in the testis was thought to confer protection against insult (Rivkees & Crawford 1988) (Fig. 2A). However, this does not appear to be the case as shown in studies demonstrating infertility in adults following childhood chemotherapy treatment (Chow *et al*. 2016).

During prepuberty, the proliferative rate of the germ cell population is reduced in comparison with foetal development; nevertheless, there is a three-fold increase in the overall germ cell population during this time period (Müller & Skakkebaek 1983). At this point, the SSC pool is represented by undifferentiated A spermatogonia composed of two populations of cells with a dark (A_dark_) or pale (A_pale_) appearance. A_dark_ spermatogonia are believed to represent the reserve stem cells with low mitotic activity, whilst the A_pale_ cells are actively proliferating. Upon differentiation, the A_pale_ spermatogonia differentiate to B spermatogonia, which can be observed from 4 to 5 years of age (Paniagua & Nistal 1984). Type B spermatogonia will enter meiosis to form spermatozoa in the adult (Ehmcke *et al*. 2006, Stukenborg *et al*. 2014). In rodents, A_single_ (A_s_) spermatogonia actively proliferate to form two conjoined daughter cells known as A_paired_ (A_pr_) cells which continuously divide forming A_aligned_ (A_al_) chains (reviewed in de Rooij & Russell 2000, Ehmcke *et al*. 2006). Occasionally, spermatogonia enter meiosis, leading to the infrequent observation of primary spermatocytes during prepuberty; however, these cells quickly degenerate since the somatic cells are not mature enough to support full spermatogenesis at this stage (Chemes 2001). The diameter of the tubules is unaltered during prepubertal development, with the lumen expanding only later during puberty due to intense germ cell proliferation that results in the expansion in tubule width (Chemes 2001). Reference values based upon a systematic review and meta-analysis performed by Masliukaite *et al.* (2016) indicates that spermatogonia number per tubular cross-section and density per area (cm^3^) decrease during the first 3 years of life followed by a gradual increase up to 6 to 7 years of age, plateauing up to age 11 when boys begin to enter puberty and numbers increase dramatically. The initial decrease may be a result of programmed cell death of the gonocytes that failed to migrate to the basement membrane earlier in development (Masliukaite *et al*. 2016).

The Sertoli cells in the prepubertal testis appear morphologically immature, with little cytoplasm and with the nuclei arranged in palisade formation with small nucleoli (Chemes 2001). These somatic cells differentiate and undergo functional maturation during prepuberty, with increased expression of androgen receptors and connexin 43, as well as expression of vimentin and inhibin β markers (Brehm *et al*. 2006, de Michele *et al*. 2018). In addition, Sertoli cells display aromatase activity and produce oestrogen during prepuberty (Chemes 2001). AMH, in particular, is secreted in large amounts in prepubertal boys and can be used as an indicator of Sertoli cell number and function; however, levels will decline during puberty and will be low throughout adulthood (Rey 1999). As in the foetus, Sertoli cell factors are involved in controlling the development/proliferation of other testicular cell types. The peritubular myoid cells proliferate and develop during early postnatal development in the rodent, under the control of FSH, with Sertoli cells having an important role in maintaining their differentiated state (Chemes 2001, Nurmio *et al*. 2012, Rebourcet *et al*. 2014). However, this dependency is lost when the peritubular myoid cells terminally differentiate during prepuberty, as shown in the rodent model (Rebourcet *et al*. 2014). These cells gain the ability to contract during puberty, with testosterone stimulating the expression of smooth muscle actin in the primate model (Mayerhofer 2013). The development of the Leydig cell population during the prepubertal period is less well understood with much of our knowledge based on rodent studies (Chen *et al*. 2009). In the rodent, Sertoli cells have an important role in stimulating ALC differentiation and will ultimately determine the number of ALCs in the adult (Rebourcet *et al*. 2014). The ALC population forms during puberty following reactivation of the HPG axis from stem/progenitor cells, which proliferate during early postnatal life (Chen *et al*. 2009).

## Evidence of chemotherapy-induced direct damage to the prepubertal testis

Understanding the specific mechanisms by which different classes of chemotherapy drugs directly target and damage the prepubertal testis is essential to aid development of protective strategies. Damage induced by chemotherapy treatment can have a major impact on the patient’s reproductive outcome in later life, with impaired development of sexual characteristics and potential fertility consequences (Frederick *et al*. 2016). Long-term fertility depends on continued survival of male germ cells, specifically SSCs, and of functional supporting somatic cells (Zohni *et al*. 2012, Yoon *et al*. 2017, Stukenborg *et al*. 2018*a*). However, research focusing on direct damage to the testis is lacking within a clinical setting since testis tissue biopsy is not routinely performed before or after chemotherapy treatment. With recent focus on cryopreserving prepubertal testis samples before the onset of cytotoxic treatment for potential fertility preservation in the future, more tissue is becoming available for research and therefore studies using such tissue should be more common in the future. Indeed, a recent report by Stukenborg *et al*. (2018*b*) has histologically examined testis biopsies from prepubertal patients who were selected for cryopreservation of tissue due to the cytotoxic nature of their cancer treatment regimens. Research from animal studies has the potential to aid in understanding gonadal toxicity of individual drugs and their mechanism of action, as well as to examine the impact of clinically relevant combined treatments; however, to date, there have been few such studies. The human and animal studies discussed in this review focus on chemotherapy treatment delivered during the prepubertal period, where the damage can be assessed after treatment or implied from subsequent analysis of the adult testis.

### Human studies

Studies examining the direct testicular damage induced following chemotherapy treatment of prepubertal human patients are few and far between, with most of these linking the damage to cyclophosphamide treatment, although in almost all cases there is co-administration of other chemotherapy drugs (Table 2). Methods of analysis are descriptive in nature following histological analysis of testicular biopsies, for example, describing the general appearance of tubules and density of germ and somatic cells present. Additional analyses include calculation of the tubular fertility index, which represents the percentage of seminiferous tubules that contain spermatogonia (Ise *et al*. 1986). To the best of our knowledge, there are no papers where chemotherapy agents were tested for cytotoxicity through *in vitro* culture of testicular cells or tissues from human patients to include in this review. Many of the included studies present limitations such as small patient cohorts, lack of universal methods for defining pubertal status and lack of adequate control groups for comparison.

**Table 2:** Human studies reporting cyclophosphamide-induced gonadotoxicity: assessment of immediate testicular damage.

Cyclophosphamide dosage	Other drugs co-administered	Treatment length	Male patients		Effect of cyclophosphamide				Reference
			*n*	Age (years)	Testicular histology	Number of germ or somatic cells/TFI	Interstitial fibrosis/basement membrane thickening	Testicular size	
Grams/kg body weight									
0.002 g/kg/day	Unknown	3 months	1 (prepubertal: 1; CYC treated: 1)	6	Abnormal, atrophic tubules	SCOs	Present	Small	Hyman and Gilbert (1972)
0.003–0.024 g/kg/day	Unknown	<50–400 days	7 (prepubertal: 7; CYC treated: 7)	3–11	Normal	N/A	N/A	N/A	Arneil (1972)
0.475–0.846 g/kg	AsparaginaseCytarabineDoxorubicinMercaptopurineMethotrexatePrednisoloneVincristine	2–6 years	46	0.08–13	Abnormal	Reduced TFI >0.130 g/kg CYC. Normal TFI intermittently treated higher cumulative CYC. Poorly developed somatic cells younger patients	N/A	N/A	Ise *et al.* (1986)
Grams/area									
0.12–8.5 g/m^2^	AsparaginaseBleomycinCarmustineCytarabineDacarbazineDactinomycinDaunorubicinDoxorubicinFluorouracilLomustineMercaptopurineMethotrexateMitomycin CNitrogen mustardPrednisoneProcarbazineTeniposideVinblastineVincristineVindesine	0.08–1 year	32 (prepubertal: 21; CYC treated: 13)	<11	Abnormal when treated with multiagent regimen including CYC	N/A	N/A	N/A	Matus‐Ridley *et al.* (1985)
0.5–1.2 g/m^2^	AsparaginaseCytarabineDaunorubicinDoxorubicinMercaptopurineMethotrexatePrednisoneVincristine	1.2–88.8 months	10 (prepubertal: 8; CYC treated: 2)	9.4–16.6	N/A	Complete loss of GCs CYC treated	N/A	N/A	Müller *et al.* (1985)
>1 g/m^2^	AsparaginaseCytarabineDoxorubicinMercaptopurineMethotrexatePrednisoloneVincristine	12–77 months	44 (prepubertal: 27)	3.5–15	N/A	Reduced TFI (<40%) >1 g/m^2^ CYC	Present	N/A	Lendon *et al.* (1979)
3–16 g/m^2^	AsparaginaseCytarabineDexamethasoneDoxorubicinMercaptopurineMethotrexatePrednisoloneThioguanineVincristine	~1.8 years	37 (prepubertal: 37; CYC treated: 16)	1.1–16.1	N/A	Depletion of spermatogonial pool with reduced TFI (19%)	N/A	N/A	Poganitsch-Korhonen *et al.* (2017)
<4.8 g/m^2^	AsparaginaseCytarabineDaunorubicinHydroxyureaLomustineMethotrexatePrednisoloneThioguanineVincristine	3–4 years	25 (prepubertal: 24)	1.23–12.35	Abnormal	Complete loss or depletion of GC pool. Normal SC	N/A	Small	Quigley *et al.* (1989)
6.2–11.4 g/m^2^	AsparaginaseCytarabineDoxorubicinMercaptopurineMethotrexatePrednisoloneTeniposideVincristine	6.6–7.6 years	23 (prepubertal: 23; CYC treated: 6)	2.8–8.6	N/A	Depletion in SSCs (↓ CD9 and OCT4) and more differentiated spermatogonia (↓ MAGE4). Recovery noted	N/A	N/A	Nurmio *et al.* (2009*a*)
8–16.8 g/m^2^	AsparaginaseCytarabineDaunorubicinDexamethasoneEnocitabineHydrocortisoneMercaptopurineMethotrexatePrednisoloneVincristine	Unknown	12 (CYC treated: 7)	1–12	N/A	Reduced TFI (<50%) with morphological changes to GCs and SCs but not linked to CYC	Present	N/A	Kobayashi *et al.* (1996)
Total dose									
1.4–20.8 g	AsparaginaseCytarabineDoxorubicinMercaptopurineMethotrexatePrednisoloneVincristine	~4.4 years	37 (CYC treated: 14)	1.6–14.3	Abnormal	GC damage and reduced TFI (<50%)	N/A	N/A	Wallace *et al.* (1991)
7.79 g	Prednisolone	180 days	1 (prepubertal: 1; CYC treated: 1)	3–4	Normal	N/A	N/A	Normal	Berry *et al.* (1972)

Studies suggest that the cumulative cyclophosphamide dose, age at treatment and patient’s sensitivity as well as the treatment regimen itself can influence the level of damage. As these patients often received a combination of chemotherapy drugs it is hard to determine the relative contributions of each drug. Studies were included only where the cyclophosphamide dosage and age of patient at time of treatment were known.

^#^Information not included in study.

CYC, cyclophosphamide; GC, germ cell; SC, Sertoli cell; SCO, Sertoli cell-only tubule; SSC, spermatogonial stem cell; TFI, tubular fertility index.

#### Assessment of immediate testicular damage in prepubertal patients

Following on from initial histological observations published in early case reports (Arneil 1972, Berry *et al*. 1972, Hyman & Gilbert 1972), larger studies indicate a relationship between use of alkylating agents in treatment regimens and testicular tissue damage (Poganitsch-Korhonen *et al*. 2017, Stukenborg *et al*. 2018*b*). In particular, the inclusion of the drug cyclophosphamide for cancer treatment during the prepubertal period has been linked to resulting testicular damage (Table 2). The studies included here under ‘immediate assessment’ varied in the timeframe of analysis with the testicular damage examined at different time points including during, just before the cessation or at the end of the treatment period as well as up to a year after the end of treatment. These investigations have indicated that cyclophosphamide treatment is associated with testicular damage in a dose- and time-dependent manner. Treatment can reduce the overall size of the testis where there is depletion of the germ cell population, resulting in Sertoli cell-only tubules, as well as interstitial fibrosis and basement membrane thickening (Hyman & Gilbert 1972, Hensle *et al*. 1984, Uderzo *et al*. 1984). A cut-off dose at which such damage is evident is hard to define, since comparison between the few available studies is challenging due to the limitations previously described, as well as the variability of treatment regimens. The length of the treatment regimens may also determine the severity of the impairment, with higher cumulative doses over a shorter period of time reducing chemotherapy-induced damage (Ise *et al*. 1986). In many of the studies listed in Table 2 and discussed further in subsequent sections, only a subset of the patients exhibit severe damage to the testis following cyclophosphamide treatment. This indicates a degree of variability/susceptibility to damage which could be due to several factors, including age and genetic predisposition.

The testis itself is composed of somatic and germ cells, which could potentially each have different sensitivities to chemotherapeutic drugs. Damage to the somatic cells during chemotherapy treatment could negatively impact on the germ cells and vice versa (Stukenborg *et al*. 2018*a*). Nurmio *et al*. (2009*a*) have reported that cyclophosphamide targets both SSCs and more differentiated spermatogonia in the prepubertal testes, as indicated by changes in gene expression of specific spermatogonial markers (MAGE A4 and CD9). This is in agreement with most papers that reported effects on germ cells, with one study describing the appearance of immature Leydig and Sertoli cells following cyclophosphamide treatment (Ise *et al*. 1986).

#### Assessment of the effect of prepubertal drug exposure damage in the adult

Insult to the prepubertal testes following chemotherapy treatment can be inferred from examination of pubertal/adult patients who were treated as children and can also determine the potential for the testes to recover and later undergo active spermatogenesis. Short-term analysis (1 to 5 years) and long-term analysis (6 to 10 years) following the cessation of cyclophosphamide treatment have shown that damage is often still observable in a dose- and time-dependent manner (Table 3). Patients receiving relatively high doses of cyclophosphamide have exhibited severe testicular damage with Sertoli cell-only tubules present up to 9 years after treatment (Penso *et al*. 1974, Aubier *et al*. 1989). The length of treatment regimen may also influence the disruption caused to the prepubertal testes, as shown in Etteldorf *et al.* (1976) (Table 3). However, such differences may ultimately be the result of higher cumulative doses or the age of the patient when treated, with younger patients potentially more at risk of reduced tubular fertility index and poor development of Sertoli and Leydig cells, as has been shown in Ise *et al.* (1986). Nonetheless, due to small numbers of participants, individual studies such as this can only lead to definitive conclusions when part of a larger meta-analysis. A case report has described somatic cell damage in the testis following chemotherapy during prepuberty, with the presence of immature Sertoli cells (identified by cytokeratin 18 and D2-40 markers) in a 31-year-old man treated during childhood with a regimen containing cyclophosphamide; however, causation cannot be determined from a case report (Bar-Shira Maymon *et al*. 2004).

**Table 3:** Human studies reporting cyclophosphamide-induced gonadotoxicity: subsequent assessment in the adult.

Cyclophosphamide dosage	Other drugs co-administered	Treatment length	Patients within the study		Effect		Reference
			*n*	Age, years	Short term (1–5 years)	Long term (6–10 years)	
Grams per body weight							
0.2–0.5 g/kg/day	Unknown	1.5–6 months	23 (prepubertal: 16; CYC treated: 23)	3.5–20	Abnormal histology 20–25% of tubules atrophic high cumulative doses	N/A	Pennisi *et al.* (1975)
0.104–0.2 g/kg	Prednisone	49–60 days	4 (CYC treated: 4)	9–13	N/A	Normal testis morphology	
0.312–1.325 g/kg	N/A	89–489 days	4 (CYC treated: 4)	5–8.5	N/A	Damage to seminiferous epithelium with SCOs	Etteldorf *et al.* (1976)
0.475–0.846 g/kg	AsparaginaseCytarabineDoxorubicinMercaptopurineMethotrexatePrednisoloneVincristine	2–6 years	46	0.08–13	Tubular damage occasionally observed 4 years after treatment with unknown ‘low’ doses	N/A	Ise *et al.* (1986)
Grams/area							
2.6–29 g/m^2^	AsparaginaseCytarabineDactinomycinDaunomycinDoxorubicinFluorouracilMechlorethamineMercaptopurineProcarbazineVincristine	Unknown	30 (prepubertal: 19; CYC treated: 10)	1.75–17	N/A	Abnormal morphology	Aubier *et al.* (1989)
2.75–7.5 g/m^2^	AsparaginaseCarmustineDoxorubicinMercaptoruineMethotrexatePrednisoneThioguanineVincristine	3–4 years	17 (prepubertal: 17; CYC treated: 5)	2.5–12.4	Reduced number of GC mm^3^	N/A	Müller *et al.* (1988)
Total dose							
0.021–39 g	FluoxymesteroneMercaptopurineOxandrolonePrednisone	9 weeks to 19 months	7 (CYC treated: 7)	11–16	High doses; SCOs, peritubular fibrosis, normal morphology LCs. Low doses; active spermatogenesis 90% of tubules	N/A	Penso *et al.* (1974)

Studies suggest that the cumulative cyclophosphamide dose, age at treatment and patient’s sensitivity as well as the treatment regimen itself can influence the level of damage. As these patients often received a combination of chemotherapy drugs it is hard to determine the relative contributions of each drug. Studies were included only where the cyclophosphamide dosage and age of patient at time of treatment were known.

CYC, cyclophosphamide; GC, germ cell; LC, Leydig cell; SCO, Sertoli cell-only tubule.

### Summary of human studies

The studies discussed above suggest that alkylating agents, in particular cyclophosphamide, can be detrimental to the prepubertal testis in a manner that can persist at least up to 10 years after the cessation of treatment. Since patients had received a combination of chemotherapy drugs, it is hard to determine the relative contributions of individual chemotherapeutic drugs to gonadal toxicity; in addition, results may have been influenced by the age/stage of pubertal development and each patient’s own sensitivity to chemotherapy treatments.

### Animal studies

The use of animal models has the potential to provide a clearer picture of chemotherapy drug-induced gonadal toxicity in the prepubertal context, in comparison to our very limited ability to investigate this directly in humans. Research on animal models enables researchers to administer drugs through more regulated regimens and to compare results using animals, tissues or cells. The use of animal models also opens the possibility of determining which period of development is more sensitive to chemotherapy treatment, for example by comparing infancy to prepuberty. Additionally, such studies are likely to be invaluable in determining the underlying mechanisms by which the different chemotherapy drugs damage the prepubertal testis, information which should help in the subsequent development of protective strategies designed to directly block such damage. Despite their great potential, relatively few studies have been conducted *in vivo* or *in vitro* with the majority of studies focusing on germ cell effects. Work to date has looked specifically at alkylating agents, anthracyclines, topoisomerase inhibitors, vinca alkaloids and non-anthracycline antibiotic chemotherapy treatments.

#### Germ cell effects

### Alkylating and alkylating-like agents

As in human studies, research to date using prepubertal testis of rodents points to alkylating and alkylating-like agents targeting the germ cell population in a dose- and time-dependent manner (Table 4). SSCs have been shown to be particularly sensitive to cyclophosphamide and cisplatin, with treatment inducing DNA damage *in vitro*, which could ultimately activate cell death pathways if not repaired by internal DNA repair systems (Marcon *et al*. 2010, Liu *et al*. 2014). DNA damage was noted following short-term (24 h) exposure to chemotherapeutics using an *in vitro* culture system of prepubertal (pnd 5) mouse testis (Smart *et al*. 2018). Activation of the apoptotic pathway was shown by enhanced cleaved caspase (CC) 3 expression following cyclophosphamide and cisplatin treatment with the timings differing between the agents, followed by loss of germ cells hours later. Increased numbers of apoptotic spermatogonia and primary spermatocytes have also been observed *in vivo* immediately following cisplatin treatment during early puberty (pnd 30–45) in the rat and after a 45-day recovery period (Lirdi *et al*. 2008, Favareto *et al*. 2011). The length of the treatment rather than the cumulative doses appears to be involved in determining the degree of testicular damage (Velez de la Calle *et al*. 1989). Shorter treatment regimens reduce time of exposure of the testis to the chemotherapeutic agent, which could be more important than overall cumulative dose received.

**Table 4:** Chemotherapy-induced gonadal toxicity – animal studies.

Chemotherapy drug	Doses (length of treatment)	Animal model (age)	*In vivo* or* in vitro*	Effect		Reference
				Germ cells	Somatic cells	
(A) Alkylating and alkylating-like agents						
Cisplatin	10–15 µg/mL	Mouse SSCs (isolated from pnd 3 to 6)	*In vitro*	↓ Survival of SSC clusters in dose-dependent manner	N/A	Shabani *et al.* (2016)
	0.1, 0.5 and 1 µg/mL (24 h)	Prepubertal mouse (pnd 5)	*In vitro*	↓ GCs, particularly SSCs↑ CC3 expression 24 h after treatment↓ GCs 8 h later ↑ γH2AX GC expression 16 h after treatment	No significant effect on SC or LC numbers	Smart *et al.* (2018)
	1 µM (48 h)	Rat SSCs (isolated from pnd 7 to 8)	*In vitro*	Dose-dependent ↑ γH2AX	N/A	Marcon *et al.* (2010)
	0.5 µg/mL (24 h)	SCs prepubertal rat (pnd 18)	*In vitro*	N/A	50% ↓ transferrin production	Nambu *et al.* (1995)
	200 or 400 µM (24 h)	SCs prepubertal rat (pnd 19)	*In vitro*	N/A	↓ Cell viability (200 µM) but no ↑ CC3 activation at 24 h	Aslani *et al.* (2017)
	0.005 g/kg	Pubertal rat (pnd 30)	*In vivo*	↓ Seminiferous epithelium ↑ Apoptotic, TUNEL positive pre-meiotic GCs and primary spermatocytes 12 h after treatment	N/A	Lirdi *et al.* (2008)
	0.001 g/kg/day (3 weeks)	Pubertal rat (pnd 45)	*In vivo*	↑ Apoptotic, TUNEL positive pre-meiotic GCs and primary spermatocytes	Vacuolation of SC↓ ABP production	Favareto *et al.* (2011)
	0.1 µM (48 h)	C18-4 spermatogonial cells (mouse)	*In vitro*	↑ γH2AX telomeres↓ Telomere length and activity of telomerase	N/A	Liu *et al.* (2014)
Cyclophosphamide	0.04–0.28 g/kg (2 weeks)	Prepubertal rat (pnd 10)	*In vivo*	No evidence of testicular damage	N/A	Velez de la Calle *et al.* (1989)
	0.002 g/kg/day (5 weeks)0.7 g/kg total	Pubertal rat (pnd 45)	*In vivo*	Atrophied seminiferous tubules↓ Spermatogonia and primary spermatocytes	Vacuolation of SC↓ ABP production	
	0.1 g/kg (10 days)	Pubertal rat (pnd 45)	*In vivo*	No evidence of testicular damage	N/A	
Cyclophosphamide (4-hydroperoxy cyclophosphamide-4OOH-CPA metabolite)	0.1 µM (48 h)	C18-4 spermatogonial cells (mouse)	*In vitro*	↑ γH2AX telomeres expression↓ Telomere length and activity reduced	N/A	Liu *et al*. (2014)
Cyclophosphamide (arcolein metabolite)	50 or 100 µM (3 or 12 h)	Mouse SCs (isolated from pnd 8)	*In vitro*	N/A	↓ Viability of SC↑ ROS and ↓ antioxidant activity↑ ApoptosisImpairs cytoskeleton of SCs	Liu *et al.* (2012)
Cyclophosphamide (phosphoramide mustard metabolite)	0.02, 0.2 and 2 µg/mL (24 h)	Prepubertal mouse (pnd 5)	*In vitro*	↓ GCs, particularly SSCs↑ CC3 expression 16 h after treatment↓ GCs 8 h later ↑ γH2AX GC expression 16 h after treatment	No effect on SC or LC numbers	Smart *et al.* (2018)
Procarbazine	0.030 g/kg/day (5 or 9 weeks)	Prepubertal (pnd 10), pubertal (pnd 45) and adult rat (pnd 70–90)	*In vivo*	5 weeks; ↓ diameter of SCO (prepubertal), less of an effect on pubertal and adults9 weeks; less impact on pubertal rats	Vacuolation of SC↓ ABP production	Velez de la Calle *et al*. (1988)
(B) Anthracyclines						
Doxorubicin	40 and 100 ng/mL (up to 72 h)	Prepubertal rat (pnd 5)	*In vitro*	↓ GC numbers 24 and 48 h↓ Proliferating and ↑ apoptotic GCs, after 16 h	No effect SCs number, production of key proteinsNo impact on PMC proliferation or LC testosterone production	Nurmio *et al*. (2009*b*)
	0.05, 0.1 and 0.5 µg/mL (24 h)	Prepubertal mouse (pnd 5)	*In vitro*	↓ GCs, particularly SSCsNo expression of CC3 observed before GC loss↑ γH2AX GC expression 16 h after treatment	No effect on SC or LC numbers	Smart *et al.* (2018)
	0.003 g/kg	Prepubertal (pnd 6 and 16) and pubertal rat (pnd 24 and 45)	*In vivo*	Prepubertal rats ↑ SSC death↓Toxicity in pubertal rats treated 3, 7 and 14 days after treatment	N/A	Bechter *et al.* (1987)
	0.003 g/kg	Prepubertal rat (pnd 6, 16 and 24)	*In vivo*	Targets migrating gonocytes↑ Apoptotic cells in pnd 6 rats 48 h after treatment↑ p53 and CC8 expression	N/A	Hou *et al.* (2005)
	0.4 µg/mL (24 h)	SCs prepubertal rat (pnd 18)	*In vitro*	N/A	35% ↓ transferrin production	Nambu *et al.* (1995)
	0.005 g/kg	Pubertal rat (pnd 22)	*In vivo*	N/A	Dysfunction and morphological alterations pnd 40, more advanced pnd 64 with recovery pnd 127 (adult)↓ Transferrin production and abnormal positioning of SC nuclei	Brilhante *et al.* (2012)
	0.1 µM (24–72 h)	GC-6 spermatogonial cell line (rat)	*In vitro*	DNA strand breaks Cell death without activation of apoptosis (externalization of phosphatidylserine)	N/A	Beaud *et al.* (2017)
	0.01–10 µM (24 h)	GC-6 spermatogonial and Ser-W3 immature SC cell line (rat)	*In vitro*	Time- and dose-dependent ↑ in cytotoxicity	Time- and dose-dependent ↑ in cytotoxicity (Ser-W3 more sensitive)↑ Oxidative stress, nuclear 8-oxo-deooxyguanosine↓ glutathione levels 6 hGlutathione supplementation did not affect survival	Tremblay and Delbes (2018)
Etoposide	1 µM (48 h)	Rat SSCs (isolated from pnd 7 to 8)	*In vitro*	↓ SSC clusters	N/A	Marcon *et al*. 2010
	25 µM or 100 µM (24 h)	SCs from prepubertal rat (pnd 19)	*In vitro*	N/A	No impact on cell viability or CC3 activation.	Aslani *et al*. 2017
	1.2 µg	Prepubertal rat (pnd 21)	*In vivo*	↑ CC9, 8 and 3 activation 24 h after treatment in spermatocytesProtein and mRNA of p53 and Bcl2 altered	N/A	Ortiz *et al.* (2009)
	0.05 g/kg/dayTotal doses 0.01, 0.02 and 0.04 g/kg	Prepubertal rat (pnd 25)	*In vivo*	Analysed pnd 26 and pnd 32↑ Apoptotic differentiated spermatogonia and primary spermatocytes except for 0.01 g/kg group when sacrificed 12 h after end of treatment	N/A	Stumpp *et al.* (2004)
	0.04 g/kg	Prepubertal rat (pnd 25)	*In vivo*	N/A	SC adluminal with chromatin clumps and vacuolization↓ Transferrin production from pnd 45 onwards	Stumpp *et al.* (2006, 2008)
	0.002 g/kg (30 days)	Pubertal rat (pnd 30)	*In vivo*	↓ GCs↑ Cell death (chromatin condensation)Damage still apparent 113 days after treatment	N/A	Freitas *et al.* (2002)
	0.01 µM (48 h)	C18-4 spermatogonial cells (mouse)	*In vitro*	No effect on levels of γH2AX levels in telomeres or telomere dysfunction	N/A	Liu *et al.* (2014)
Irinotecan (SN38 metabolite)	0.1 and 1 µg/mL (24 h)	Prepubertal rat (pnd 5)	*In vitro*	Targets the proliferating germ cell population	N/A	Lopes *et al.* (2016)
(D) Vinca alkaloids and antibiotics						
Bleomycin	0.1 µM (48 h)	Rat SSCs (isolated from pnd 7 to 8)	*In vitro*	↓ Cluster number and area in culture	N/A	Marcon *et al.* (2010)
	1.5 µM (48 h)	C18-4 spermatogonial cells (mouse)	*In vitro*	↑ DNA damage in the telomeres, no impact on telomerase activity	N/A	Liu *et al.* (2014)
Mitomycin C	500 µg/kg (alternate day for 20 days)	Pubertal rats (pnd 40)	*In vivo*	N/A	↓ Leydig cell nuclear area↓ 3β-Hydroxysteroid dehydrogenase	Deb *et al.* (1980)
Vincristine	0.1 µM (48 h)	Rat SSCs (isolated from pnd 7 to 8)	*In vitro*	Targets SSC in a dose-dependent manner	N/A	Marcon *et al.* (2010)
	0.5 µg/mL (24 h)	SCs from prepubertal rat (pnd 18)	*In vitro*	N/A	↓ Transferrin production	Nambu *et al.* (1995)
	0.1 µM (24–72 h)	GC-6 spermatogonial cell line (rat)	*In vitro*	↓ Cell viability↑ Cell death differentiated spermatogonia dose- and time-dependent mannerNo observable DNA damage but activation of apoptotic pathways	N/A	Beaud *et al.* (2017)
(E) Combination treatments						
Bleomycin, cisplatin and etoposide	0.1 µM (48 h)	Rat SSCs (isolated from pnd 7 to 8)	*In vitro*	Combination had no additional impact on cluster size/area of SSCs	N/A	Marcon *et al.* (2010)
Vincristine and doxorubicin	0.01 µM (24–72 h)	GC-6 spermatogonial cell line (rat)	*In vitro*	Combination ↑ cell death of spermatogonia dose-dependent manner in comparison to individual treatment	N/A	Beaud *et al.* (2017)

Gonadotoxicity determined with different classes either in isolation or combination. Results suggest that chemotherapy-induced damage is dependent on the chemotherapeutic agent, cumulative dose, stage of development and treatment regimen.

ABP, androgen binding protein; CC, cleaved caspase; GC, germ cell; LC, Leydig cell; PMC, peritubular myoid cell; pnd, postnatal day; SC, Sertoli cell; SCO, Sertoli cell-only tubule; SSCs, spermatogonial stem cells.

### Anthracyclines

Of the anthracycline class of drugs, work to date has focused on doxorubicin, and it is clear that this drug targets the pre-mitotic dividing spermatogonia and the pre-meiotic primary spermatocytes in the prepubertal testis (Table 4). These germ cells are undergoing DNA synthesis and therefore contain high levels of the enzyme topoisomerase II, which is a target of the drug (Parvinen & Parvinen 1978). During early prepubertal development, the testis is especially vulnerable to doxorubicin-induced damage, depleting the seminiferous epithelium in comparison to later stages shown in the rat model (Bechter *et al*. 1987). This study found using an immature *in vivo* rat model, that a relatively low dose of doxorubicin (3 mg/kg) was not sufficient to kill all the SSCs, as some recovery was apparent. In addition to inhibition of topoisomerase II activity, doxorubicin has been shown to induce DNA damage in spermatogonia* in vitro*, which can result in cell death (Beaud *et al*. 2017). Interestingly, the Beaud *et al*. (2017) work indicated that cell death was induced independent of apoptosis, since externalization of phosphotidylerine was not apparent following treatment. In agreement with these findings, DNA damage was observed despite the absence of a significant increase in CC3 expression prior to loss of germ cells following short-term exposure of prepubertal mouse testis tissue to doxorubicin (Smart *et al*. 2018). The cell death that occurs following doxorubicin may result from a non-apoptotic mechanism for example through necrosis or autophagy (Beaud *et al*. 2017, Smart *et al*. 2018). This hypothesis would be consistent with studies analysing cardiotoxicity following doxorubicin treatment where the cell death induced is through autophagy (Dirks-Naylor 2013). However, increased levels of CC8 and p53 48 h after treatment in an *in vivo* model has also been reported (Hou *et al*. 2005). Oxidative stress has also been proposed as a mechanism of doxorubicin-induced damage to germ cells, however, work in an *in vitro* model culturing a cell line with rat SSC/spermatogonia type A characteristics (GC-6spg) has shown no such increase in levels of reactive oxygen species (ROS) before the onset of cytotoxicity (Tremblay & Delbes 2018).

### Topoisomerase inhibitors

Topoisomerase inhibitors, such as etoposide and irinotecan, have been investigated to determine prepubertal gonadal toxicity and have been found to target the pre-mitotic and pre-meiotic germ cells (Table 4). These drugs inhibit the activity of the enzyme topoisomerase I and II and ultimately induce cell death through activation of apoptotic pathways (Freitas *et al*. 2002, Stumpp *et al*. 2004, Ortiz *et al*. 2009). Etoposide damages the prepubertal testis depleting the germ cell pool, with little recovery from treatment at low doses (2 mg/kg; Freitas *et al*. 2002). SSCs are particularly vulnerable to etoposide treatment, with a lower half maximal inhibitory dose in comparison to cisplatin and bleomycin. With etoposide targeting the SSC population, this reduction could account for the reduction in later stages of spermatogenesis observed in the adult rat (Stumpp *et al*. 2004, Marcon *et al*. 2010). Exposure of the prepubertal mouse testis *in vitro* to concentrations of SN38 (the metabolite of irinotecan) that reflect patient serum levels shows that this drug targets the proliferating germ cell population (Lopes *et al*. 2016). Involvement of the apoptotic pathway in cell death has been observed following etoposide treatment in an *in vivo* prepubertal rat model, with increased numbers of apoptotic intermediary and type B spermatogonia, as well as primary spermatocytes, immediately following treatment (Stumpp *et al*. 2004). In addition, increased activity of CC9, CC3 and CC8 as well as enhanced levels of protein and mRNA of p53 and Bcl-2 have been observed in the prepubertal rat following etoposide treatment (Ortiz *et al*. 2009). However, from this study it is not possible to determine which cell types within the testis the observed changes occurred in, since results were obtained from homogenised tissue.

### Vinca alkaloids and non-anthracycline antibiotics

*In vitro* studies indicate that vincristine may target the germ cell population and bleomycin has the potential to damage the testis (Table 4). Vincristine reduces cell viability and increases cell death of the GC-6spg cell line in a dose- and time-dependent manner. However, this was not a result of DNA damage, as this class of drugs inhibits polymerization of microtubules, involving activation of apoptotic pathways (Beaud *et al*. 2017). Bleomycin targets SSCs *in vitro* in a dose-dependent fashion, with cytotoxicity (seen at 0.1 µM) inducing DNA damage that extends into telomere regions of chromosomes (Marcon *et al*. 2010, Liu *et al*. 2014). These initial studies indicate that such drugs have the potential to impact the testis negatively and should be further investigated *in vivo*.

### Combination treatments

In a clinical setting, cancer is treated with a combination of chemotherapeutic agents. Different classes of drugs target cells through differing mechanisms which may have synergistic effects. The combined treatments reduce the chance of resistance and survival of cancerous cells, but have the possibility of leading to multiplicative off-target side effects on healthy cells. Relatively few animal studies have focused on combinations of drugs (Table 4). Using the GC-6spg cell line, Beaud *et al*. (2017) showed that a combination of vincristine and doxorubicin enhanced levels of cell death in a dose-dependent manner in comparison to treatment with each drug individually. In contrast, exposure to a combination of bleomycin, cisplatin and etoposide had no additional impact on cluster size/area of mouse SSCs in culture (Marcon *et al*. 2010). Further investigations into common combinations are required to determine the relative gonadotoxicity, and whether the effects are synergistic and/or multiplicative.

#### Somatic cell effects

Studies relating to impacts of chemotherapy treatment upon the somatic cells have mainly been limited to the Sertoli cell and include studies which have focused on cyclophosphamide, cisplatin, doxorubicin and etoposide chemotherapy agents (Table 4). Treatment results in morphological damage including vacuolation of the cells and adluminal positioning (Velez de la Calle *et al*. 1988,^ 1989^, Stumpp *et al*. 2006,^ 2008^, Favareto *et al*. 2011, Brilhante *et al*. 2012). However, these cells do survive exposure as shown in Smart *et al.* (2018) where there was no overall change in cell numbers following cyclophosphamide, cisplatin or doxorubicin treatment in an *in vitro* model of prepubertal mouse testicular tissue. Damage to the Sertoli cells resulting in dysfunction could be the primary effect of such treatment, which would have a significant impact on testis function. Indeed, decreased production of androgen-binding protein following treatment with alkylating and alkylating-like agents has been reported in prepubertal rats (Velez de la Calle *et al*. 1988,^ 1989^, Favareto *et al*. 2011). *In vitro* studies using primary cultures of rat Sertoli cells have also shown reduced transferrin production following cisplatin, doxorubicin and vincristine treatment (Nambu *et al*. 1995). Transferrin stimulates germ cell proliferation/differentiation by transferring iron to these cells (Sylvester & Griswold 1994). A potential mechanism by which cyclophosphamide and doxorubicin induce damage specifically to the Sertoli cell has been proposed by *in vitro* studies of cultured immature Sertoli cells where enhanced levels of oxidative stress have been reported (Liu *et al*. 2012, Tremblay & Delbes 2018) as well as damage to the cytoskeleton following cyclophosphamide treatment (Liu *et al*. 2012).

Impairment of Sertoli cell functionality, however, may be secondary and a result of primary injury to the germ cell population. An *in vivo* study looking at doxorubicin-induced damage has shown that the alterations in the morphology and function of these cells was more pronounced in early adulthood in comparison to time of treatment just before puberty (pnd 22). This suggests that the germ cells were the primary cells targeted by doxorubicin, with loss/damage to the germ cells ultimately impacting Sertoli cells as a secondary effect (Brilhante *et al*. 2012). For etoposide, functional deficits were also more apparent in adulthood, suggesting damage was secondary to germ cell death. However, upon recovery of the seminiferous epithelium, Sertoli cell dysfunction was still apparent with reduced transferrin production and altered morphology; this may suggest a degree of primary damage on the Sertoli cells (Stumpp *et al*. 2006). How to distinguish between primary and secondary damage to the Sertoli cells is difficult due to the dependence of Sertoli cells on germ cells and vice versa. Isolation of Sertoli cells from the germ cell population, as with the primary cell culture or established cell line culture experiments, has been used to look specifically at Sertoli cell damage. These isolated Sertoli cells, however, may not be representative of the ‘true’ *in vivo* Sertoli cell as often these cells do not maintain Sertoli cell identity once removed from their true environment, a limitation that needs to be borne in mind for all cell line studies.

To date, few studies have focused on the other somatic cell types within testis, the Leydig cells or peritubular myoid cells. An *in vivo* study reported reduced steroidogenic activity of Leydig cells following treatment of immature rats with mitomycin C (Deb *et al*. 1980). More recent *in vitro* studies have shown there was no change in Leydig cells numbers reported by Smart *et al*. (2018) after *in vitro* exposure of mouse prepubertal testis fragments to cisplatin, cyclophosphamide and doxorubicin. The proliferative ability of the peritubular myoid cells and steroidogenic activity of the Leydig cells were also unaltered after doxorubicin treatment of rat testis tissue *in vitro* (Nurmio *et al*. 2009*b*). Additional studies looking specifically at these cell types within the testis are required before any conclusion can be drawn on chemotherapy-induced damage.

### Summary of animal studies

To conclude, in the prepubertal testes chemotherapy agents have been shown to specifically target and deplete the germ cell pool, in some cases specifically the SSC population, with DNA damage noted after cyclophosphamide, cisplatin and doxorubicin exposure. Apoptosis is the main cell death pathway activated by cyclophosphamide, cisplatin, etoposide and vincristine exposure, whereas doxorubicin-induced testicular damage may be the result of an alternative cell death pathway such as necrosis or autophagy. Cancer therapy may also affect the Sertoli cell population resulting in morphological damage and/or dysfunction, as shown by cyclophosphamide, procarbazine, cisplatin, doxorubicin and etoposide treatment. However, somatic cell impairment may either be the result of primary cellular damage to the Sertoli cells themselves and/or a secondary consequence of targeted loss of the germ cell population. Whether combined chemotherapy treatment regimens modify the toxicity of individual drugs needs further clarification, as few such *in vitro* studies have been performed to date.

## Effects on future generations

The clinical impact of prepubertal chemotherapy treatment on later fertility has been discussed in several reviews (Hudson 2010, Lee & Shin 2013); for survivors who are able to conceive there could be potential effects upon future generations due to unrepaired damage to the male germline. The impact on future generations is not yet clear. A Danish study of 472 survivors of childhood cancer found no significant association between alkylating chemotherapy treatment and later genetic diseases of the progeny of these survivors (Winther *et al*. 2012). Nonetheless, a study by Liu *et al*. (2014) has shown a potential mechanism by which alkylating agents can impact on future generations by targeting the telomeres of mouse spermatogonial cells. Telomerase function was reduced at concentrations of a drug precursor of cyclophosphamide (4OOH-CPA) and cisplatin which induced significant spermatogonial cell death resulting in reduced telomere length and activity of telomerase. A reduction in the length or function of telomeres of the male germ cell can adversely affect early development of the offspring, increasing the rate of pre- and/or post-implantation loss, congenital malformation and miscarriage (Liu *et al*. 2002). Whether additional classes of drugs also impact on future generations is unknown and therefore further research into this area is urgently required.

## Protective strategies

The development of chemotherapeutic treatment regimens has increased greatly in the past decade resulting in a greater number of childhood cancer survivors reaching adulthood and facing long-term consequences of treatment, such as infertility. There is therefore an increasing focus on preserving the fertility of children undergoing cancer treatment. At the time of writing, though, fertility preservation strategies for prepubertal boys have yet to be established clinically. Testicular tissue from boys undergoing ‘high’ risk chemotherapy treatment is being collected in a limited number of centres, with tissue cryopreserved and stored for potential fertility restoration when the boys reach adulthood. The proposed techniques to restore fertility, however, are still in the experimental phase of development with success to date only in rodent models (Picton *et al*. 2015, Giudice *et al*. 2017). Studies with human tissues have been conducted in relation to transplantation of cryopreserved testicular tissue and SSCs as well as *in vitro* maturation of immature testicular tissue, paving the way for the development of a fertility restoration method clinically (reviewed Giudice *et al*. 2017). Xenotransplantation of prepubertal human testicular tissue for up to 9 months has been performed with nude mice where initiation of spermatogenesis was observed within the grafted tissue, however spermatogenesis did not reach meiotic differentiation (Wyns *et al*. 2007,^ 2008^, Goossens *et al*. 2008, Van Saen *et al*. 2011,^ 2013^). SSCs isolated from prepubertal patients have been propagated *in vitro*, but results are preliminary (Sadri-Arekani *et al*. 2011). Human prepubertal testicular tissue has been successfully cultured *in vitro* with survival of the spermatogonial germ cell population, maturation of the somatic cells and formation of the blood–testis barrier reported (de Michele *et al*. 2017,^2018^). Initiation of spermatogenesis from such tissue has yet to be demonstrated. Alternatively, the prepubertal testis could be protected against injury caused by the treatment through use of cytoprotective agents that would be added to the cancer treatment regimen. Several studies have shown the gonadoprotective potential of agents in animal studies focusing on adult males, mainly investigating the morphology and motility of sperm. For example, Carmely *et al*. (2009) has shown in a mouse model the cytoprotective effect of the immunomodulator AS101 compound against cyclophosphamide-induced testicular damage. In contrast, so far only a limited number of compounds have been analysed to determine their ability to protect the prepubertal testis in an animal model (Table 5).

**Table 5:** Potential cytoprotective agents to protect the prepubertal testis against chemotherapy-induced damage.

Compound	Dose	Chemotherapy drug/doses	Animal model/age	*In vivo* or *in vitro*	Effect	Reference
Amifostine	0.2 g/kg	Doxorubicin 0.003 g/kg	Prepubertal rat (pnd 6)	*In vivo*	No protective effects	Jahnukainen *et al.* (2001), Hou *et al.* (2005)
Amifostine	0.4 g/kg	Cisplatin 0.005 g/kg	Prepubertal rat (pnd 30)	*In vivo*	Partial protection, ↓ seminiferous tubule area and ↑ apoptotic spermatogonia and primary spermatocytes	Lirdi *et al.* (2008)
Amifostine	0.4 g/kg	Doxorubicin 0.005 g/kg	Prepubertal rat (pnd 30)	*In vivo*	Partially protects. Did not protect against DNA damage and negatively impacted on embryo development and pregnancy outcome	Vendramini *et al.* (2010, 2012)
Amifostine	1 µM	Doxorubicin 0.01–1 µM (24 h)	GC-6 spermatogonial and Ser-W3 immature SC cell line (rat)	*In vitro*	Pre-treatment for 24 h or co-treatment had no impact on cytotoxicity in the Ser-W3 cell line	Tremblay & Delbes (2018)
Cartinine	0.25 g/kg	Etoposide 0.04 g/kg	Prepubertal rat (pnd25)	*In vivo*	Analysed pnd 30, 64 and 100. Partial protection, reduction in TUNEL+ cells	Okada *et al.* (2009)
Cartinine	0.25 g/kg/day	Doxorubicin 0.005 g/kg	Prepubertal rat (pnd30)	*In vivo*	Analysed pnd 64 and 100. Partial protection, ↓ TUNEL+ cells and sperm DNA damage. ↑ Acrosome integrity pre-treatment, no impact on sperm motility and mitochondrial activity. ↓ Lipid peroxidation and nitric oxide. ↑ Fertility index and implantation rate improved	Cabral *et al.* (2014, 2018)
Cartinine	10 mM	Doxorubicin 0.01–1 µM (24 h)	GC-6 spermatogonial and Ser-W3 immature SC cell line (rat)	*In vitro*	Pre-treatment for 24 h or co-treatment had no impact on cytotoxicity in the Ser-W3 cell line	Tremblay & Delbes (2018)
Curcumin	5 µM	Doxorubicin 0.01–1 µM (24 h)	GC-6 spermatogonial and Ser-W3 immature SC cell line (rat)	*In vitro*	Pre-treatment for 24 h or co-treatment had no impact on cytotoxicity in the Ser-W3 cell line	Tremblay & Delbes (2018)
Ginseng intestinal metabolite I (GIM-I)	0.05 g/kg/day	Doxorubicin 0.05 g/kg/day	Prepubertal rat (pnd28)	*In vivo*	Partially protects	Kang *et al.* (2002)
Vitamin C	40 µg/mL	Doxorubicin 0.01–1 µM (24 h)	GC-6 spermatogonial and Ser-W3 immature SC cell line (rat)	*In vitro*	Pre-treatment for 24 h or co-treatment had no impact on cytotoxicity in the Ser-W3 cell line	Tremblay & Delbes (2018)

### Amifostine

Amifostine is an organic thiophosphate that acts as a cytoprotective agent, protecting cells against chemotherapeutic damage whilst having no antitumor activity (Çetingül *et al*. 2009). The active metabolite acts as a ROS scavenger and binds and stabilizes DNA (Spencer & Goa 1995). This drug has a limited half-life of eight minutes, therefore *in vivo* studies have focused on pre-treatment of amifostine 15 min before chemotherapy treatment. Pre-treatment partially protected the prepubertal rat testis against cisplatin- and doxorubicin-induced testicular damage; this effect may be dependent on the age and schedule of treatment, as a lower dose of amifostine in earlier stages of prepubertal development in rats had no protective effects against doxorubicin-induced damage when tissues were analysed 24 and 48 h after treatment (Jahnukainen *et al*. 2001, Hou *et al*. 2005). However, prepubertal amifostine pre-treatment before doxorubicin treatment did not maintain fertility in the adult, as DNA damage was found in the sperm of treated animals when analysed 64 days after treatment, which increased the number of arrested embryos in a mating study of adults (100 days old) (Vendramini *et al*. 2012). An *in vitro* study has also indicated that amifostine has no protective effects against doxorubicin-induced damage in a spermatogonial cell line and an immature Sertoli cell line (Tremblay & Delbes 2018). Therefore, amifostine may not be a suitable fertility preservation strategy.

### Carnitine

Carnitine is a quaternary amine found at high concentrations within the epididymis of the male reproductive tract and in spermatozoa. It is acquired through dietary meat and milk and is also produced by the liver through methylation of lysine and methionine amino acids. This compound has essential roles in determining male fertility, producing energy by transferring long-chain fatty acids into mitochondria, a process required for germ cell maturation, sperm motility and sperm count; it has been shown to have cytoprotective properties whilst having no impact on anticancer treatment efficacy (Chiu *et al*. 2004, Sayed-Ahmed 2010). Partial protection from etoposide and doxorubicin-induced morphological damage and apoptotic germ cell death was shown when prepubertal rats were pre-treated with carnitine one hour before chemotherapy treatment *in vivo* (Okada *et al*. 2009, Cabral *et al*. 2014). The mechanism underlying such cytoprotective action is unknown, but may be the result of enhanced DNA repair activity, inhibition of ceramide production and/or reduction in oxidative stress-induced damage as shown in a range of different cell types (Andrieu-Abadie *et al*. 1999, Palmero *et al*. 2000, Alshabanah *et al*. 2010). Indeed, a study by Cabral *et al.* (2018) has shown improved oxidative stress status of the adult testis following prepubertal pre-treatment with cartinine one hour before doxorubicin treatment in a rodent model. An *in vitro* model, however, has found no protective effects against doxorubicin-induced damage when cartinine is administered in spermatogonial or immature Sertoli cell lines (Tremblay & Delbes 2018). Sertoli cell function may be improved upon carnitine administration, as these cells express carnitine/organic cation transport 2 receptors, with carnitine important in the maintenance of the BTB (Palmero *et al*. 2000).

### Ginseng intestinal metabolite I (GIM-I)

The herbal root, Ginseng has been used in East Asian countries as a traditional Chinese medicine and the intestinal metabolite, known as ginseng intestinal metabolite I (GIM-I), is thought to have multiple pharmacological effects through its antioxidant activity (Zhang *et al*. 1996). The protective effect against doxorubicin-induced damage has been analysed by Kang *et al*. (2002) in prepubertal mice *in vivo*, where GIM-I was found to partially protect against doxorubicin-induced germ cell damage resulting in testicular morphology comparable to controls that may be the result of enhanced antioxidant activity. GIM-I increases the levels of testis-specific antioxidants which were reduced following doxorubicin treatment. This compound has great potential as a cytoprotective agent against doxorubicin-induced damage and appears to have anti-metastatic activity and could therefore be added to chemotherapeutic regimens to provide benefit as both a cytotoxic and cytoprotective agent (Hasegawa *et al*. 1997).

### Vitamin C and curcumin

A study by Tremblay and Delbes (2018) has investigated the potential of compounds as cytoprotectants based upon their antioxidant activity, including vitamin C and curcumin. Vitamin C can also function by inhibiting apoptosis, whilst curcumin has additional anti-inflammatory properties. However, in both cases treatment did not reduce the cytotoxic activity of doxorubicin in either spermatogonial or immature Sertoli cell lines. This study therefore indicates that these compounds may not be suitable cytoprotectants against doxorubicin-induced prepubertal testicular damage.

### Summary of protective strategies

Cytoprotective agents could play a major role in the future of fertility preservation strategies, however, research in this area to date is very limited, and has only shown partial protection against chemotherapy-induced damage with pre-treatment with amifostine, cartinine and GIM-I. Moreover, prepubertal amifostine treatment alone compromised later fertility and is therefore not suitable for purpose. In contrast, GIM-I might have more potential as a cytoprotective agent, in part due to its anti-metastatic activity. Overall, despite the promising results outlined, the level of evidence established so far in animal studies in not sufficient for transfer to clinical practise and needs further investigation.

## Conclusion

This review has provided an overview of what is currently known in relation to chemotherapy-induced prepubertal testicular toxicity from studies in human patients and animal models, focusing primarily upon direct damage following chemotherapy exposure. Cancer therapy with a range of chemotherapy agents from different drug classes during childhood have been found to negatively impact upon the prepubertal testis. The resulting damage depends on the compounds used, cumulative dose, administration regimen and age/pubertal status during treatment. Such conclusions have been drawn from both clinical investigations and animal models, including *in vivo* as well as *in vitro* studies, with testicular fragments or primary cell cultures/cell lines representatives of the cell types in the prepubertal testis. However, as this review has discussed, the evidence for chemotherapy-induced damage to the prepubertal testis is at present incomplete and needs further investigation.

Further research into chemotherapy-induced prepubertal testicular toxicity is essential as the number of childhood cancer survivors is set to increase steadily over the coming years. Enhancing our knowledge of the gonadotoxicity of chemotherapeutic agents is essential for clinicians to determine which patients to offer cryopreservation of immature testicular tissue for potential fertility restoration strategies, which are at present experimental for human patients. Understanding how chemotherapy agents target and damage the testis of young boys will provide much clarity to the future quality of life of these patients and aid in the development of protective strategies for preservation of fertility.

## Declaration of interest

Norah Spears is a member of the Editorial Board of *Reproduction*. None of the other authors has any conflict of interest.

## Funding

Funding by Children with Cancer UK (grant #15-198). R M’s work was undertaken in the MRC Centre for Reproductive Health funded by MRC Centre Grant MR/N022556/1, and C A was supported by Career Development PhD Scholarship in Biomedical Sciences funded by the Biomedical Sciences ZJU project. Thanks to Kathleen Duffin for comments on an earlier draft.
